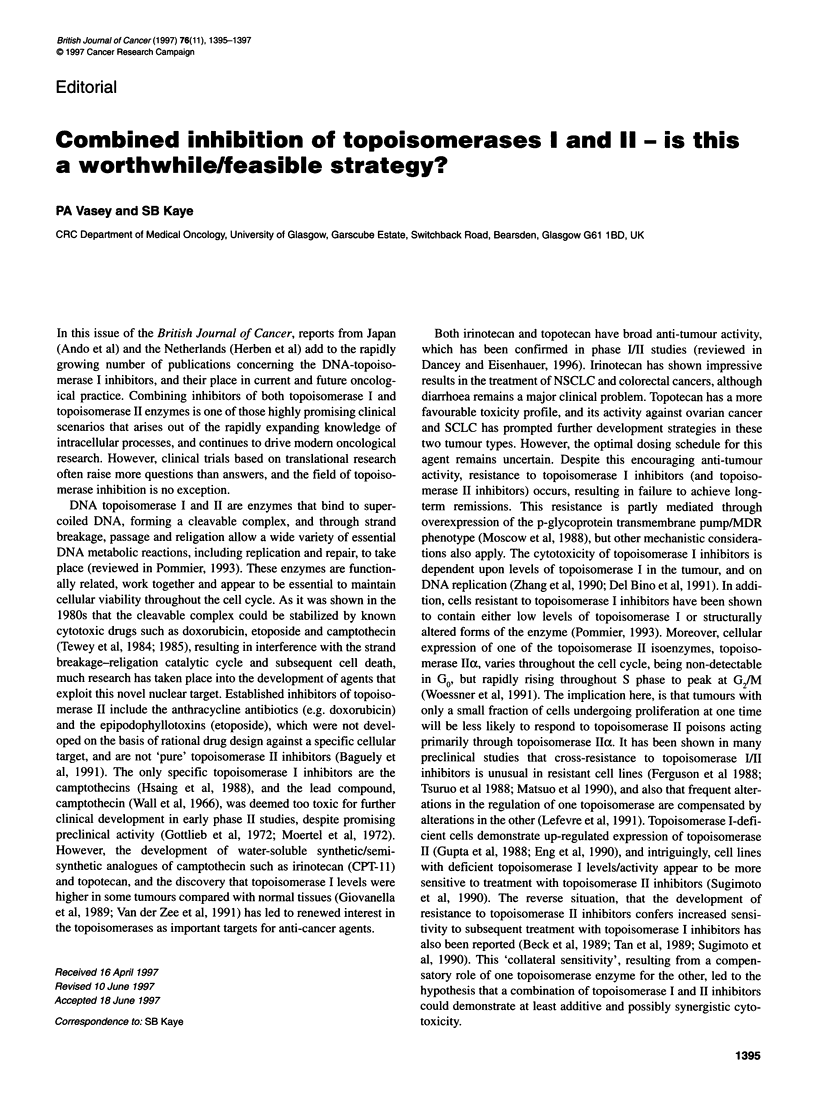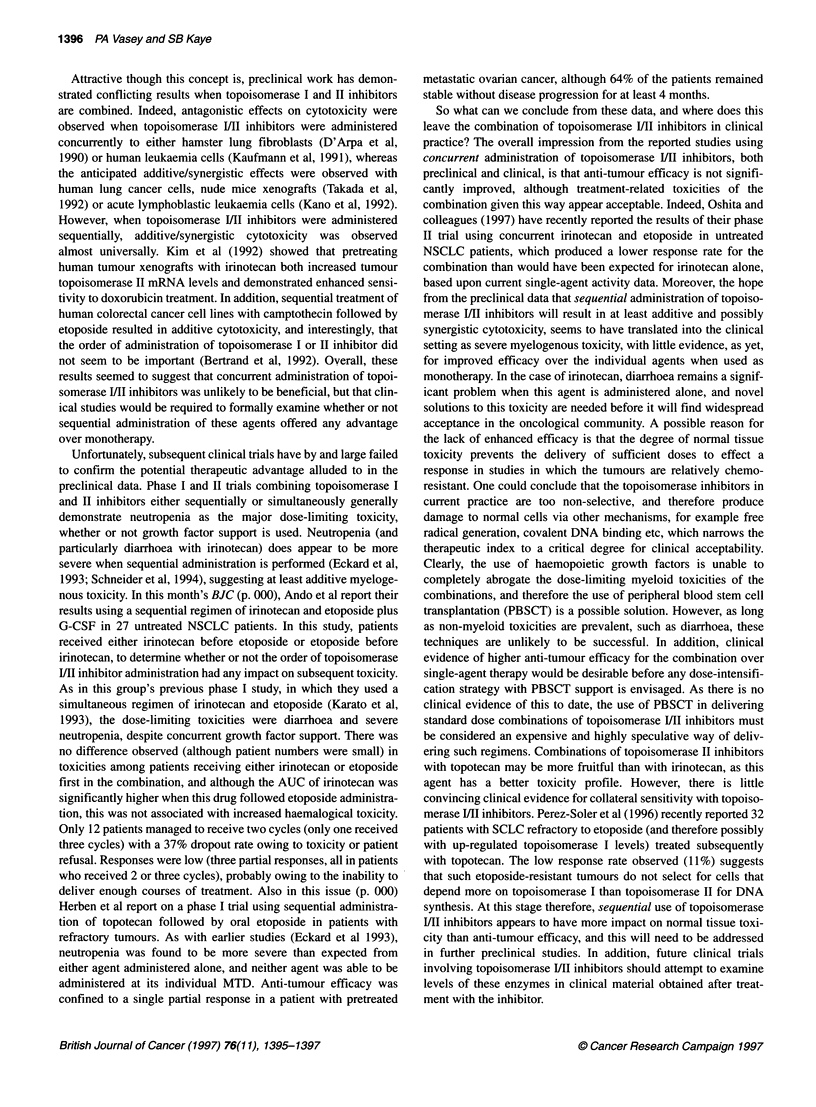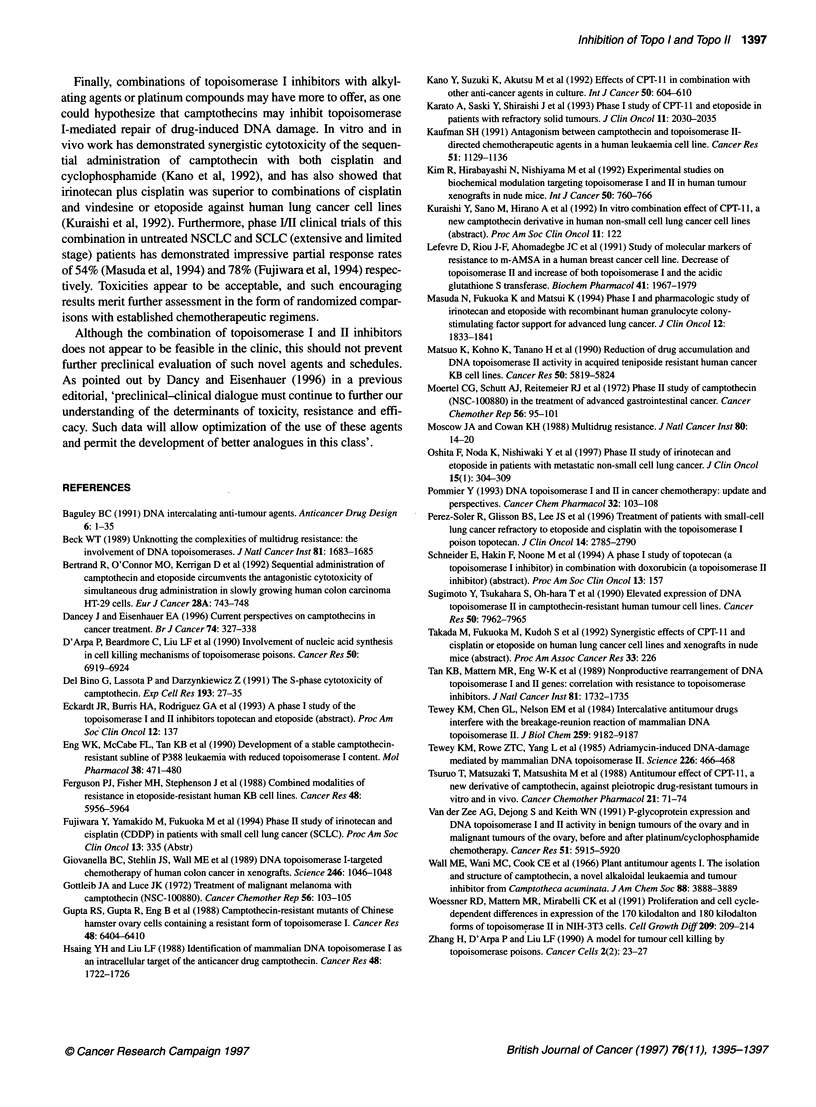# Combined inhibition of topoisomerases I and II--is this a worthwhile/feasible strategy?

**DOI:** 10.1038/bjc.1997.568

**Published:** 1997

**Authors:** P. A. Vasey, S. B. Kaye


*British Journal of Cancer* (1997) **76**(11), 1395–1397
© 1997 Cancer Research Campaign

Editorial

### Combined inhibition of topoisomerases I and II – is this a worthwhile/feasible strategy?

**PA Vasey and SB Kaye**

CRC Department of Medical Oncology, University of Glasgow, Garscube Estate, Switchback Road, Bearsden, Glasgow G61 1BD, UK

In this issue of the *British Journal of Cancer*, reports from Japan (Ando et al) and the Netherlands (Herben et al) add to the rapidly growing number of publications concerning the DNA-topoisomerase I inhibitors, and their place in current and future oncological practice. Combining inhibitors of both topoisomerase I and topoisomerase II enzymes is one of those highly promising clinical scenarios that arises out of the rapidly expanding knowledge of intracellular processes, and continues to drive modern oncological research. However, clinical trials based on translational research often raise more questions than answers, and the field of topoisomerase inhibition is no exception.

DNA topoisomerase I and II are enzymes that bind to supercoiled DNA, forming a cleavable complex, and through strand breakage, passage and religation allow a wide variety of essential DNA metabolic reactions, including replication and repair, to take place (reviewed in Pommier, 1993). These enzymes are functionally related, work together and appear to be essential to maintain cellular viability throughout the cell cycle. As it was shown in the 1980s that the cleavable complex could be stabilized by known cytotoxic drugs such as doxorubicin, etoposide and camptothecin (Tewey et al, 1984; 1985), resulting in interference with the strand breakage–religation catalytic cycle and subsequent cell death, much research has taken place into the development of agents that exploit this novel nuclear target. Established inhibitors of topoisomerase II include the anthracycline antibiotics (e.g. doxorubicin) and the epipodophyllotoxins (etoposide), which were not developed on the basis of rational drug design against a specific cellular target, and are not 'pure' topoisomerase II inhibitors (Baguely et al, 1991). The only specific topoisomerase I inhibitors are the camptothecins (Hsaing et al, 1988), and the lead compound, camptothecin (Wall et al, 1966), was deemed too toxic for further clinical development in early phase II studies, despite promising preclinical activity (Gottlieb et al, 1972; Moertel et al, 1972). However, the development of water-soluble synthetic/semi-synthetic analogues of camptothecin such as irinotecan (CPT-11) and topotecan, and the discovery that topoisomerase I levels were higher in some tumours compared with normal tissues (Giovanella et al, 1989; Van der Zee et al, 1991) has led to renewed interest in the topoisomerases as important targets for anti-cancer agents.

*Received 16 April 1997*
*Revised 10 June 1997*
*Accepted 18 June 1997*

*Correspondence to:* SB Kaye

Both irinotecan and topotecan have broad anti-tumour activity, which has been confirmed in phase I/II studies (reviewed in Dancey and Eisenhauer, 1996). Irinotecan has shown impressive results in the treatment of NSCLC and colorectal cancers, although diarrhoea remains a major clinical problem. Topotecan has a more favourable toxicity profile, and its activity against ovarian cancer and SCLC has prompted further development strategies in these two tumour types. However, the optimal dosing schedule for this agent remains uncertain. Despite this encouraging anti-tumour activity, resistance to topoisomerase I inhibitors (and topoisomerase II inhibitors) occurs, resulting in failure to achieve long-term remissions. This resistance is partly mediated through overexpression of the p-glycoprotein transmembrane pump/MDR phenotype (Moscow et al, 1988), but other mechanistic considerations also apply. The cytotoxicity of topoisomerase I inhibitors is dependent upon levels of topoisomerase I in the tumour, and on DNA replication (Zhang et al, 1990; Del Bino et al, 1991). In addition, cells resistant to topoisomerase I inhibitors have been shown to contain either low levels of topoisomerase I or structurally altered forms of the enzyme (Pommier, 1993). Moreover, cellular expression of one of the topoisomerase II isoenzymes, topoisomerase IIα, varies throughout the cell cycle, being non-detectable in $G_0$, but rapidly rising throughout S phase to peak at $G_2$/M (Woessner et al, 1991). The implication here, is that tumours with only a small fraction of cells undergoing proliferation at one time will be less likely to respond to topoisomerase II poisons acting primarily through topoisomerase IIα. It has been shown in many preclinical studies that cross-resistance to topoisomerase I/II inhibitors is unusual in resistant cell lines (Ferguson et al 1988; Tsuruo et al 1988; Matsuo et al 1990), and also that frequent alterations in the regulation of one topoisomerase are compensated by alterations in the other (Lefevre et al, 1991). Topoisomerase I-deficient cells demonstrate up-regulated expression of topoisomerase II (Gupta et al, 1988; Eng et al, 1990), and intriguingly, cell lines with deficient topoisomerase I levels/activity appear to be more sensitive to treatment with topoisomerase II inhibitors (Sugimoto et al, 1990). The reverse situation, that the development of resistance to topoisomerase II inhibitors confers increased sensitivity to subsequent treatment with topoisomerase I inhibitors has also been reported (Beck et al, 1989; Tan et al, 1989; Sugimoto et al, 1990). This 'collateral sensitivity', resulting from a compensatory role of one topoisomerase enzyme for the other, led to the hypothesis that a combination of topoisomerase I and II inhibitors could demonstrate at least additive and possibly synergistic cytotoxicity.

Attractive though this concept is, preclinical work has demonstrated conflicting results when topoisomerase I and II inhibitors are combined. Indeed, antagonistic effects on cytotoxicity were observed when topoisomerase I/II inhibitors were administered concurrently to either hamster lung fibroblasts (D'Arpa et al, 1990) or human leukaemia cells (Kaufmann et al, 1991), whereas the anticipated additive/synergistic effects were observed with human lung cancer cells, nude mice xenografts (Takada et al, 1992) or acute lymphoblastic leukaemia cells (Kano et al, 1992). However, when topoisomerase I/II inhibitors were administered sequentially, additive/synergistic cytotoxicity was observed almost universally. Kim et al (1992) showed that pretreating human tumour xenografts with irinotecan both increased tumour topoisomerase II mRNA levels and demonstrated enhanced sensitivity to doxorubicin treatment. In addition, sequential treatment of human colorectal cancer cell lines with camptothecin followed by etoposide resulted in additive cytotoxicity, and interestingly, that the order of administration of topoisomerase I or II inhibitor did not seem to be important (Bertrand et al, 1992). Overall, these results seemed to suggest that concurrent administration of topoisomerase I/II inhibitors was unlikely to be beneficial, but that clinical studies would be required to formally examine whether or not sequential administration of these agents offered any advantage over monotherapy.

Unfortunately, subsequent clinical trials have by and large failed to confirm the potential therapeutic advantage alluded to in the preclinical data. Phase I and II trials combining topoisomerase I and II inhibitors either sequentially or simultaneously generally demonstrate neutropenia as the major dose-limiting toxicity, whether or not growth factor support is used. Neutropenia (and particularly diarrhoea with irinotecan) does appear to be more severe when sequential administration is performed (Eckard et al, 1993; Schneider et al, 1994), suggesting at least additive myelogenous toxicity. In this month's *BJC* (p. 000), Ando et al report their results using a sequential regimen of irinotecan and etoposide plus G-CSF in 27 untreated NSCLC patients. In this study, patients received either irinotecan before etoposide or etoposide before irinotecan, to determine whether or not the order of topoisomerase I/II inhibitor administration had any impact on subsequent toxicity. As in this group's previous phase I study, in which they used a simultaneous regimen of irinotecan and etoposide (Karato et al, 1993), the dose-limiting toxicities were diarrhoea and severe neutropenia, despite concurrent growth factor support. There was no difference observed (although patient numbers were small) in toxicities among patients receiving either irinotecan or etoposide first in the combination, and although the AUC of irinotecan was significantly higher when this drug followed etoposide administration, this was not associated with increased haemalogical toxicity. Only 12 patients managed to receive two cycles (only one received three cycles) with a 37% dropout rate owing to toxicity or patient refusal. Responses were low (three partial responses, all in patients who received 2 or three cycles), probably owing to the inability to deliver enough courses of treatment. Also in this issue (p. 000) Herben et al report on a phase I trial using sequential administration of topotecan followed by oral etoposide in patients with refractory tumours. As with earlier studies (Eckard et al 1993), neutropenia was found to be more severe than expected from either agent administered alone, and neither agent was able to be administered at its individual MTD. Anti-tumour efficacy was confined to a single partial response in a patient with pretreated metastatic ovarian cancer, although 64% of the patients remained stable without disease progression for at least 4 months.

So what can we conclude from these data, and where does this leave the combination of topoisomerase I/II inhibitors in clinical practice? The overall impression from the reported studies using *concurrent* administration of topoisomerase I/II inhibitors, both preclinical and clinical, is that anti-tumour efficacy is not significantly improved, although treatment-related toxicities of the combination given this way appear acceptable. Indeed, Oshita and colleagues (1997) have recently reported the results of their phase II trial using concurrent irinotecan and etoposide in untreated NSCLC patients, which produced a lower response rate for the combination than would have been expected for irinotecan alone, based upon current single-agent activity data. Moreover, the hope from the preclinical data that *sequential* administration of topoisomerase I/II inhibitors will result in at least additive and possibly synergistic cytotoxicity, seems to have translated into the clinical setting as severe myelogenous toxicity, with little evidence, as yet, for improved efficacy over the individual agents when used as monotherapy. In the case of irinotecan, diarrhoea remains a significant problem when this agent is administered alone, and novel solutions to this toxicity are needed before it will find widespread acceptance in the oncological community. A possible reason for the lack of enhanced efficacy is that the degree of normal tissue toxicity prevents the delivery of sufficient doses to effect a response in studies in which the tumours are relatively chemoresistant. One could conclude that the topoisomerase inhibitors in current practice are too non-selective, and therefore produce damage to normal cells via other mechanisms, for example free radical generation, covalent DNA binding etc, which narrows the therapeutic index to a critical degree for clinical acceptability. Clearly, the use of haemopoietic growth factors is unable to completely abrogate the dose-limiting myeloid toxicities of the combinations, and therefore the use of peripheral blood stem cell transplantation (PBSCT) is a possible solution. However, as long as non-myeloid toxicities are prevalent, such as diarrhoea, these techniques are unlikely to be successful. In addition, clinical evidence of higher anti-tumour efficacy for the combination over single-agent therapy would be desirable before any dose-intensification strategy with PBSCT support is envisaged. As there is no clinical evidence of this to date, the use of PBSCT in delivering standard dose combinations of topoisomerase I/II inhibitors must be considered an expensive and highly speculative way of delivering such regimens. Combinations of topoisomerase II inhibitors with topotecan may be more fruitful than with irinotecan, as this agent has a better toxicity profile. However, there is little convincing clinical evidence for collateral sensitivity with topoisomerase I/II inhibitors. Perez-Soler et al (1996) recently reported 32 patients with SCLC refractory to etoposide (and therefore possibly with up-regulated topoisomerase I levels) treated subsequently with topotecan. The low response rate observed (11%) suggests that such etoposide-resistant tumours do not select for cells that depend more on topoisomerase I than topoisomerase II for DNA synthesis. At this stage therefore, *sequential* use of topoisomerase I/II inhibitors appears to have more impact on normal tissue toxicity than anti-tumour efficacy, and this will need to be addressed in further preclinical studies. In addition, future clinical trials involving topoisomerase I/II inhibitors should attempt to examine levels of these enzymes in clinical material obtained after treatment with the inhibitor.

© Cancer Research Campaign 1997

Finally, combinations of topoisomerase I inhibitors with alkylating agents or platinum compounds may have more to offer, as one could hypothesize that camptothecins may inhibit topoisomerase I-mediated repair of drug-induced DNA damage. In vitro and in vivo work has demonstrated synergistic cytotoxicity of the sequential administration of camptothecin with both cisplatin and cyclophosphamide (Kano et al, 1992), and has also showed that irinotecan plus cisplatin was superior to combinations of cisplatin and vindesine or etoposide against human lung cancer cell lines (Kuraishi et al, 1992). Furthermore, phase I/II clinical trials of this combination in untreated NSCLC and SCLC (extensive and limited stage) patients has demonstrated impressive partial response rates of 54% (Masuda et al, 1994) and 78% (Fujiwara et al, 1994) respectively. Toxicities appear to be acceptable, and such encouraging results merit further assessment in the form of randomized comparisons with established chemotherapeutic regimens.

Although the combination of topoisomerase I and II inhibitors does not appear to be feasible in the clinic, this should not prevent further preclinical evaluation of such novel agents and schedules. As pointed out by Dancy and Eisenhauer (1996) in a previous editorial, 'preclinical–clinical dialogue must continue to further our understanding of the determinants of toxicity, resistance and efficacy. Such data will allow optimization of the use of these agents and permit the development of better analogues in this class'.

### REFERENCES

Baguley BC (1991) DNA intercalating anti-tumour agents. *Anticancer Drug Design* **6**: 1–35

Beck WT (1989) Unknotting the complexities of multidrug resistance: the involvement of DNA topoisomerases. *J Natl Cancer Inst* **81**: 1683–1685

Bertrand R, O'Connor MO, Kerrigan D et al (1992) Sequential administration of camptothecin and etoposide circumvents the antagonistic cytotoxicity of simultaneous drug administration in slowly growing human colon carcinoma HT-29 cells. *Eur J Cancer* **28A**: 743–748

Dancey J and Eisenhauer EA (1996) Current perspectives on camptothecins in cancer treatment. *Br J Cancer* **74**: 327–338

D'Arpa P, Beardmore C, Liu LF et al (1990) Involvement of nucleic acid synthesis in cell killing mechanisms of topoisomerase poisons. *Cancer Res* **50**: 6919–6924

Del Bino G, Lassota P and Darzynkiewicz Z (1991) The S-phase cytotoxicity of camptothecin. *Exp Cell Res* **193**: 27–35

Eckardt JR, Burris HA, Rodriguez GA et al (1993) A phase I study of the topoisomerase I and II inhibitors topotecan and etoposide (abstract). *Proc Am Soc Clin Oncol* **12**: 137

Eng WK, McCabe FL, Tan KB et al (1990) Development of a stable camptothecin-resistant subline of P388 leukaemia with reduced topoisomerase I content. *Mol Pharmacol* **38**: 471–480

Ferguson PJ, Fisher MH, Stephenson J et al (1988) Combined modalities of resistance in etoposide-resistant human KB cell lines. *Cancer Res* **48**: 5956–5964

Fujiwara Y, Yamakido M, Fukuoka M et al (1994) Phase II study of irinotecan and cisplatin (CDDP) in patients with small cell lung cancer (SCLC). *Proc Am Soc Clin Oncol* **13**: 335 (Abstr)

Giovanella BC, Stehlin JS, Wall ME et al (1989) DNA topoisomerase I-targeted chemotherapy of human colon cancer in xenografts. *Science* **246**: 1046–1048

Gottleib JA and Luce JK (1972) Treatment of malignant melanoma with camptothecin (NSC-100880). *Cancer Chemother Rep* **56**: 103–105

Gupta RS, Gupta R, Eng B et al (1988) Camptothecin-resistant mutants of Chinese hamster ovary cells containing a resistant form of topoisomerase I. *Cancer Res* **48**: 6404–6410

Hsaing YH and Liu LF (1988) Identification of mammalian DNA topoisomerase I as an intracellular target of the anticancer drug camptothecin. *Cancer Res* **48**: 1722–1726

Kano Y, Suzuki K, Akutsu M et al (1992) Effects of CPT-11 in combination with other anti-cancer agents in culture. *Int J Cancer* **50**: 604–610

Karato A, Saski Y, Shiraishi J et al (1993) Phase I study of CPT-11 and etoposide in patients with refractory solid tumours. *J Clin Oncol* **11**: 2030–2035

Kaufman SH (1991) Antagonism between camptothecin and topoisomerase II-directed chemotherapeutic agents in a human leukaemia cell line. *Cancer Res* **51**: 1129–1136

Kim R, Hirabayashi N, Nishiyama M et al (1992) Experimental studies on biochemical modulation targeting topoisomerase I and II in human tumour xenografts in nude mice. *Int J Cancer* **50**: 760–766

Kuraishi Y, Sano M, Hirano A et al (1992) In vitro combination effect of CPT-11, a new camptothecin derivative in human non-small cell lung cancer cell lines (abstract). *Proc Am Soc Clin Oncol* **11**: 122

Lefevre D, Riou J-F, Ahomadegbe JC et al (1991) Study of molecular markers of resistance to m-AMSA in a human breast cancer cell line. Decrease of topoisomerase II and increase of both topoisomerase I and the acidic glutathione S transferase. *Biochem Pharmacol* **41**: 1967–1979

Masuda N, Fukuoka K and Matsui K (1994) Phase I and pharmacologic study of irinotecan and etoposide with recombinant human granulocyte colony-stimulating factor support for advanced lung cancer. *J Clin Oncol* **12**: 1833–1841

Matsuo K, Kohno K, Tanano H et al (1990) Reduction of drug accumulation and DNA topoisomerase II activity in acquired teniposide resistant human cancer KB cell lines. *Cancer Res* **50**: 5819–5824

Moertel CG, Schutt AJ, Reitemeier RJ et al (1972) Phase II study of camptothecin (NSC-100880) in the treatment of advanced gastrointestinal cancer. *Cancer Chemother Rep* **56**: 95–101

Moscow JA and Cowan KH (1988) Multidrug resistance. *J Natl Cancer Inst* **80**: 14–20

Oshita F, Noda K, Nishiwaki Y et al (1997) Phase II study of irinotecan and etoposide in patients with metastatic non-small cell lung cancer. *J Clin Oncol* **15**(1): 304–309

Pommier Y (1993) DNA topoisomerase I and II in cancer chemotherapy: update and perspectives. *Cancer Chem Pharmacol* **32**: 103–108

Perez-Soler R, Glisson BS, Lee JS et al (1996) Treatment of patients with small-cell lung cancer refractory to etoposide and cisplatin with the topoisomerase I poison topotecan. *J Clin Oncol* **14**: 2785–2790

Schneider E, Hakin F, Noone M et al (1994) A phase I study of topotecan (a topoisomerase I inhibitor) in combination with doxorubicin (a topoisomerase II inhibitor) (abstract). *Proc Am Soc Clin Oncol* **13**: 157

Sugimoto Y, Tsukahara S, Oh-hara T et al (1990) Elevated expression of DNA topoisomerase II in camptothecin-resistant human tumour cell lines. *Cancer Res* **50**: 7962–7965

Takada M, Fukuoka M, Kudoh S et al (1992) Synergistic effects of CPT-11 and cisplatin or etoposide on human lung cancer cell lines and xenografts in nude mice (abstract). *Proc Am Assoc Cancer Res* **33**: 226

Tan KB, Mattern MR, Eng W-K et al (1989) Nonproductive rearrangement of DNA topoisomerase I and II genes: correlation with resistance to topoisomerase inhibitors. *J Natl Cancer Inst* **81**: 1732–1735

Tewey KM, Chen GL, Nelson EM et al (1984) Intercalative antitumour drugs interfere with the breakage-reunion reaction of mammalian DNA topoisomerase II. *J Biol Chem* **259**: 9182–9187

Tewey KM, Rowe ZTC, Yang L et al (1985) Adriamycin-induced DNA-damage mediated by mammalian DNA topoisomerase II. *Science* **226**: 466–468

Tsuruo T, Matsuzaki T, Matsushita M et al (1988) Antitumour effect of CPT-11, a new derivative of camptothecin, against pleiotropic drug-resistant tumours in vitro and in vivo. *Cancer Chemother Pharmacol* **21**: 71–74

Van der Zee AG, Dejong S and Keith WN (1991) P-glycoprotein expression and DNA topoisomerase I and II activity in benign tumours of the ovary and in malignant tumours of the ovary, before and after platinum/cyclophosphamide chemotherapy. *Cancer Res* **51**: 5915–5920

Wall ME, Wani MC, Cook CE et al (1966) Plant antitumour agents I. The isolation and structure of camptothecin, a novel alkaloidal leukaemia and tumour inhibitor from *Camptotheca acuminata. J Am Chem Soc* **88**: 3888–3889

Woessner RD, Mattern MR, Mirabelli CK et al (1991) Proliferation and cell cycle-dependent differences in expression of the 170 kilodalton and 180 kilodalton forms of topoisomerase II in NIH-3T3 cells. *Cell Growth Diff* **209**: 209–214

Zhang H, D'Arpa P and Liu LF (1990) A model for tumour cell killing by topoisomerase poisons. *Cancer Cells* **2**(2): 23–27

© Cancer Research Campaign 1997